# High‐Throughput Formation of Pre‐Vascularized hiPSC‐Derived Hepatobiliary Organoids on a Chip via Nonparenchymal Cell Grafting

**DOI:** 10.1002/advs.202407945

**Published:** 2025-01-04

**Authors:** Han Fan, Jia Shang, Junbo Li, Bo Yang, Ding Zhou, Shanqing Jiang, Yuhang Fan, Ying Zhou, Yuwen Wang, Peidi Liu, Changyong Li, Zhishui Chen, Pu Chen

**Affiliations:** ^1^ Tissue Engineering and Organ Manufacturing (TEOM) Lab Department of Biomedical Engineering Wuhan University TaiKang Medical School (School of Basic Medical Sciences) Wuhan 430071 China; ^2^ Department of Biological Repositories Zhongnan Hospital of Wuhan University Wuhan 430071 China; ^3^ Key Laboratory of Organ Transplantation Institute of Organ Transplantation Tongji Hospital Tongji Medical College Huazhong University of Science and Technology Wuhan 430030 China; ^4^ Research Center for Medicine and Structural Biology of Wuhan University Wuhan University Wuhan Hubei 430071 China; ^5^ Department of Physiology Wuhan University TaiKang Medical School (School of Basic Medical Sciences) Wuhan Hubei 430071 China; ^6^ TaiKang Center for Life and Medical Sciences Wuhan University Wuhan 430071 China

**Keywords:** hepatobiliary organoids on a chip, microfabricated hexagonal closely packed cavity arrays, nonparenchymal cell grafting, pre‐vascularization

## Abstract

Liver organoids have been increasingly adopted as a critical in vitro model to study liver development and diseases. However, the pre‐vascularization of liver organoids without affecting liver parenchymal specification remains a long‐lasting challenge, which is essential for their application in regenerative medicine. Here, the large‐scale formation of pre‐vascularized human hepatobiliary organoids (vhHBOs) is presented without affecting liver epithelial specification via a novel strategy, namely nonparenchymal cell grafting (NCG). Endothelial and mesenchymal cells are grafted to human hepatobiliary organoids (hHBOs) at the different liver epithelial differentiation stages without supplementing with nonparenchymal culture medium and growth factors. Endothelial grafting at the stage of hepatic maturation offers an optimal integration efficiency compared to the stage of hepatic specification. Additionally, grafting with mesenchymal proves crucial in endothelial invading and sprouting into the liver epithelial cells during the establishment of vhHBOs. Ectopic liver implants into mice further displayed integration of vhHBOs into mice vascular networks. Notably, transplanted vhHBOs self‐organized into native liver tissue like hepatic zone and bile ducts, indicating their potential to regenerate damaged hepatic and bile duct tissues. It is believed that nonparenchymal cell grafting will offer a novel technical route to form a high‐fidelity complex in vitro model for tissue engineering and regenerative medicine.

## Graphical abstract

This study presents an innovative approach to pre‐vascularize hPSC‐derived organoids while maintaining parenchymal fate specification, enabling in vivo transplantation and tissue fusion. Notably, transplanted pre‐vascularized human hepatobiliary organoids (vhHBOs) self‐organize into native liver tissue like hepatic zone and bile ducts, indicating their potential to regenerate damaged hepatic and bile duct tissues. This nonparechymal cell grafting strategy is expected to be broadly applicable beyond hepatobiliary organoids.

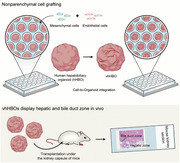

## 1 Introduction

Liver organoids represent a competitive in vitro model system for human liver development and homeostasis studies and have been increasingly utilized in human liver disease modeling, hepatotoxicity assessment, and new drug discovery.^[^ ^1^, ^2^, ^3^, ^4^, ^5^ ^]^ For example, human liver organoids have been demonstrated to emulate pivotal pathophysiological features of diverse liver diseases and facilitate mechanistic studies of the onset and progression of viral hepatitis,^[^ ^6^, ^7^ ^]^ metabolic‐associated fatty liver disease,^[^ ^8^, ^9^, ^10^ ^]^ and liver fibrosis.^[^ ^11^ ^]^ Liver organoids are stem cell‐derived 3D cell aggregates comprising liver parenchymal cells with or without supporting cells.^[^ ^12^, ^13^ ^]^ Specifically, adult stem cells are limited to generating liver organoids containing only liver epithelial cells. In contrast, pluripotent stem cells enable the generation of liver organoids containing both parenchymal and non‐parenchymal cells. Despite the successful generation of Kupffer cells and hepatic stellate cells in the liver organoids via co‐differentiation strategy from a homogeneous human pluripotent stem cells (hPSCs) population, the generation of liver organoid with endothelial cells remains an unfilled task.^[^ ^13^ ^]^ However, it is critical for diverse applications such as advanced liver models for liver organogenesis and liver regenerative therapy.

To address the issue of generating liver organoids with endothelial cells, several strategies have been proposed. One notable strategy involves coculturing liver parenchymal cells (such as hepatic endoderm,^[^ ^14^, ^15^ ^]^ hepatocyte‐like cells,^[^ ^16^, ^17^ ^]^ and primary hepatocytes)^[^ ^18^ ^]^ with endothelial cells. For example, Takebe et al. demonstrated the coculture of human induced pluripotent stem cell (hiPSC)‐derived hepatic endoderm, endothelial cells, and septum transversum cells.^[^ ^14^ ^]^ Pettino et al. also demonstrated the coculture of hiPSCs with human adipose microvascular endothelial cells.^[^ ^19^ ^]^ However, the coculture strategy often fails to generate liver organoids with complex hierarchical organization due to nonparenchymal cell culture factors affecting liver parenchymal differentiation and homeostasis. Another strategy involves co‐differentiating stem cells into liver‐specific and vascular lineages. Following this approach, Harrison et al. developed vascularized liver organoids by driving hPSC aggregates to mesodermal differentiation before initiating liver differentiation.^[^ ^20^ ^]^ However, the co‐differentiate strategy faces challenges in controlling the proportions of endothelial cells and their maturation status because mesodermal cells develop randomly.

Here, we develop an nonparenchymal cell grafting (NCG) strategy to facilitate the construction of pre‐vascularized human hepatobiliary organoids (vhHBOs) with invading endothelial cells via nonparenchymal cell grafting without supplementing nonparenchymal culture factors. Particularly, we generated human foregut stem cells (hFSCs) from hiPSCs and then differentiated hFSCs spheroids into human hepatobiliary organoids (hHBOs) in a microfabricated hexagonal closely packed cavity array (mHCPCA) chip without Matrigel embedding. We investigated the endothelial grafting efficiency during the varied differentiation stages of liver epithelium. Additionally, we examined the effects of mesenchymal grafting to facilitate endothelial sprouting and invasion into the liver epithelium. Finally, we performed ectopic vhHBO implants in mice and investigated the integration of vhHBOs in the mouse.

## 2 Result

### 2.1 mHCPCAs for High‐Throughput Production of hHBOs

**Figure 1:**
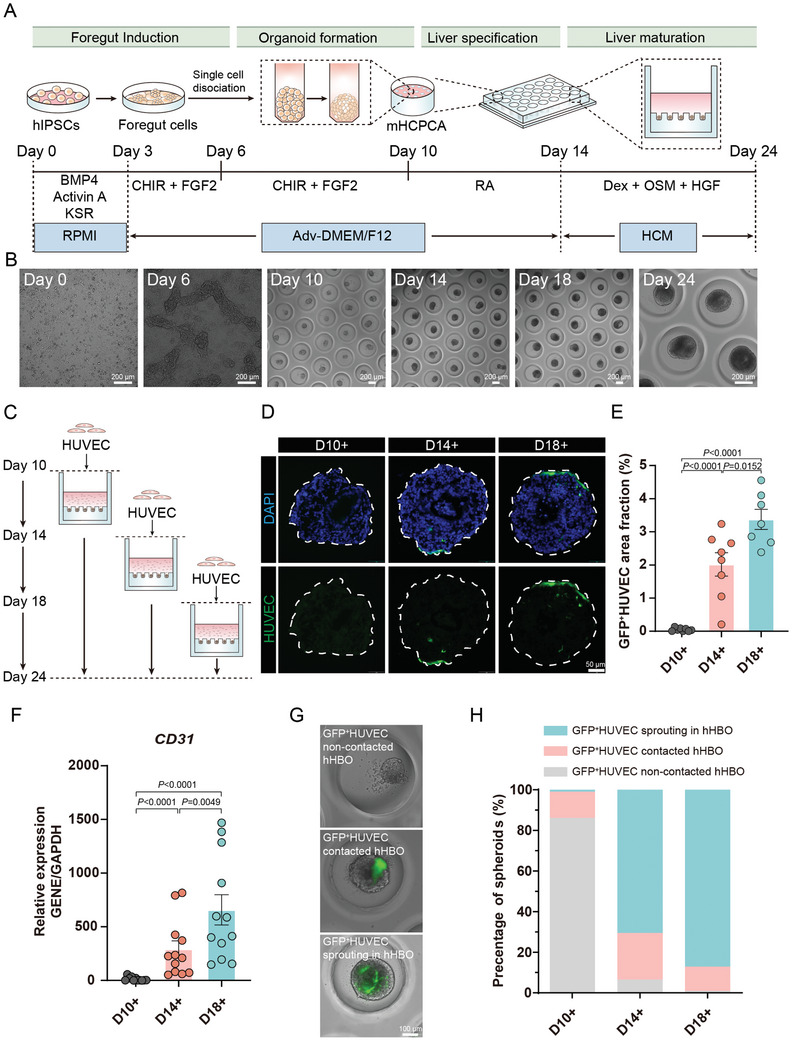
Investigation of endothelial grafting for vhHBOs. A) Schematic illustration of hiPSC differentiation into hHBOs highlights the four main differentiation stages. Full details in methods. B) Real‐time microscopy images displaying hHBOs in the mHCPCAs on days 10, 14, 18, and 24. Scale bar, 200 µm. C) Experimental design of HUVECs grafting at hHBOs during differentiation. D) Comparative morphological analysis of area fraction of GFP^+^HUVEC in each organoid section at different stages (days 10, 14 18). Scale bar, 50 µm. E) Quantification of the proportion of GFP^+^HUVEC area fraction in each organoid section. The results are shown as the mean ± SEM of 4 independent experiments. F) qRT‐PCR analysis of endothelial‐specific marker CD31 expression on day 24 under the indicated growth condition. The results are shown as the mean ± SEM of 3 independent experiments (each experiment contains 4 technical replicates). P values were calculated by unpaired two‐tailed Student's *t*‐test or one‐way ANOVA. G) Images of GFP^+^HUVECs‐to‐hHBOs integration exhibiting GFP^+^HUVEC non‐contacted organoid (top), GFP^+^HUVEC contacted organoid (middle) and GFP^+^HUVEC sprouting in organoid (bottom). Scale bar, 50 µm. H) Quantifying positional relationships of GFP^+^HUVEC to hHBO in three distinct groups at the endpoint of the culture, with results from *n* = 3 independent experiments.

We fabricated a mHCPCA chip for high‐throughput and reproducible generation of hHBOs in a Matrigel‐free environment, following the method previously described.^[^ ^21^ ^]^ The mHCPCA chips were embedded in the 48‐well plate, with each well enabling the formation of 170 liver organoids. Specifically, the hiPSCs were initially differentiated into hFSCs in the monolayer (2D) culture. On day 6, immunofluorescence analysis indicates nearly most of the cells were positive for foregut endoderm markers SOX17 and SOX2 (Figure S1, Supporting Information).^[^ ^22^ ^]^ Then, the hFSCs were detached from the plate and seeded to mHCPCAs at an average seeding density of 200 cells per microcavity. During the first 48 h, cells were sedimented by gravity into individual microcavities and then continued to aggregate to form solid hFSCs spheroids. At 96 h, the medium was switched to liver specification media, and the liver specification step was triggered, resulting in the generation of hepatoblast spheroids. Finally, the media was switched to liver maturation medium for 10 days for further maturation of hepatoblast spheroids (**Figure** **1**A,B). Image analysis indicated that each microwell contained a single hHBO and that the average maximum diameter of the hHBOs was ≈315 µm at day 24 (Figure S2, Supporting Information).

### 2.2 Grafting Endothelial Cells at the Hepatic Maturation Stage Promotes Pre‐Vascularization

We added GFP^+^HUVECs to hFSC spheroids (day 10) and immature hHBOs (days 14 and 18) (Figure 1C). Fluorescent images demonstrated that the group of day 18 derived hHBOs showed a higher green fluorescence intensity than the group of day 10 derived hHBOs or the group of day 14 derived hHBOs (Figure S3, Supporting Information). Then, we detected the mRNA level of the endothelial cell marker *CD31*, the results showed that the mRNA expression of *CD31* in group of day 14 derived hHBOs (601.7% ± 117.5%) and the group of day 18 derived hHBOs (1261.3% ± 123.6%) was upregulated by 18 and 38 times, compared to the group of day 10 derived hHBOs (33.3% ± 8.6%), respectively (Figure 1F). Moreover, we measured the area fraction of GFP^+^HUVEC in each organoid section. Similarly, the percentage of GFP^+^HUVEC coverage was significantly increased in endothelial grafting on day 18 group (3.38 ± 0.30%) than endothelial grafting on day 10 and 14 groups (0.05 ± 0.02%; 2.02 ± 0.35%) (Figure 1D,E). These results indicated that GFP^+^HUVECs could survive in the liver organoid differentiation medium without endothelial cell supplements, and grafting GFP^+^HUVECs on day 18 enabled optimal endothelial integration efficiency in the hHBOs.

We classified vhHBOs into three types, including GFP^+^HUVEC sprouting in hHBOs, GFP^+^HUVEC contacted hHBOs, and GFP^+^HUVEC non‐contacted hHBOs, based on the phenotypes of integrated GFP^+^HUVEC in the organoid. We noted that these three architectural arrangements correlated with the timing of endothelial cells grafting to hHBOs. Unsurprisingly, the percentage of GFP^+^HUVEC sprouting hHBOs was significantly increased in the group of day 18 derived hHBOs (87.0 ± 11.2%) than the group of day 14 derived hHBOs (70.5 ± 2.0%) or the group of day 10 derived hHBOs (0.97 ± 0.97%). On the contrary, the percentage of GFP^+^HUVEC non‐contacted hHBOs was found to be decreased in the group of day 18 derived hHBOs (0.97% ± 0.97%) compared with the group of day 14 derived hHBOs (6.5% ± 5.2%) or the group of day 10 derived hHBOs (0.97% ± 0.97%) (Figure 1G,H). These results suggested that the endothelial grafting at the stage of hepatic maturation offers an optimal integration efficiency compared to the stage of hepatic specification.

Additionally, we investigated the effects of endothelial grafting on hepatic specification. Immunofluorescence analysis of vhHBOs on day 24 indicated a similar protein expression pattern across the endothelial grafting groups and the control group. They were positive for hepatocyte‐specific markers ALB, AFP, and HNF4α, and all contained a liver stem cell niche (Co‐expressing EpCAM, AFP, CK19, and ALB) (Figure S3, Supporting Information). Collectively, adding GFP^+^HUVECs generated functional hHBOs similar to the control group. These results indicated that adding endothelial cells on day 18 promotes endothelial cell survival and integration with hHBOs, further promoting the pre‐vascularization of the hHBOs.

### 2.3 Mesenchymal Cells Grafting Decreasing Phenotypic Heterogeneity of vhHBOs in Size and Roundness

**Figure 2:**
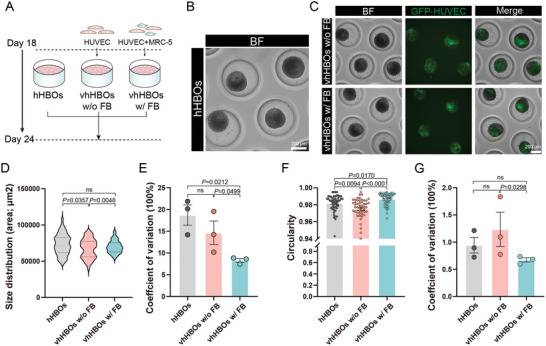
Investigation of mesenchymal grafting for vhHBOs morphology. A) Experimental design of organoid in three different culture configurations, hHBOs, vhHBOs w/o FB, or vhHBOs w/ FB. B) Representative bright‐field images of hHBOs cultures on day 24. Scale bar, 200 µm. C) The gross morphology demonstrating GFP^+^HUVEC integration within vhHBOs from the w/o FB and w/ FB groups, was captured using both bright field and fluorescent imaging techniques. Scale bar, 200 µm. D) Violin plot illustrating the distribution of organoid areas on Day 24 across the three culture configurations. The pink dotted line represents the mean, while the black dotted lines represent the quartiles. Graph represents mean ± SEM of *n* = 50 biological replicates from *n* = 3 independent experiments. P values were calculated by unpaired two‐tailed Student's *t*‐test or one‐way ANOVA. E) Coefficient of variation for the organoid area within each experimental condition. Graphs show individual data points derived from *n* = 3 independent experiments and means ± SEM. P values were calculated by unpaired two‐tailed Student's *t*‐test or one‐way ANOVA. F) Organoid circularity measurements on Day 24 from the three culture setups. Graph represents mean ± SEM of *n* = 50 biological replicates from *n* = 3 independent experiments. P values were calculated by unpaired two‐tailed Student's *t*‐test or one‐way ANOVA. G) The coefficient of variation in the circularity of organoids in each condition. Graphs show individual data points derived from *n* = 3 independent experiments and means ± SEM. P values were calculated by unpaired two‐tailed Student's *t*‐test or one‐way ANOVA.

We investigated whether the addition of mesenchymal cells would affect the organoid homogeneity. We tested three different culture conditions, mono‐culture with only hHBOs (referred to as the “hHBOs” group), a combination of hHBOs with HUVECs only (the “vhHBOs w/o FB” group), and a combination of hHBOs with HUVECs and fibroblasts (the “vhHBOs w/ FB” group) (**Figure** **2**A). Brightfield and GFP fluorescence images were taken repeatedly to observe the development of both hHBOs and endothelial networks (Figure 2B,C). The morphology of the hHBOs was assessed through brightfield image analysis; the vhHBOs w/ FB group exhibited low variation in both size and circularity compared to the vhHBOs w/o FB or the hHBOs group. Specifically, the CVs for organoid size in the vhHBOs w/ FB group were lower than the CVs in the other two conditions (8.282% ± 0.4336% versus 18.73% ± 2.329% of the hHBOs group, p = 0.02121; 8.28% ± 0.4336% versus 14.64% ± 2.711% of vhHBOs w/o FB group, p = 0.0499 (Figure 2D,E). Similarly, the CVs for organoid circularity in the vhHBOs w/ FB group were lower than the CVs in the vhHBOs w/o FB group (8.282% ± 0.4336% versus 18.73% ± 2.329% (Figure 2G). Additionally, adding mesenchymal cells increased the circularity of the organoids (vhHBOs w/ FB, vhHBOs w/o FB) (Figure 2F).

### 2.4 Mesenchymal Cells Grafting Promotes Endothelial Cells Invading and Sprouting into the hHBOs

**Figure 3:**
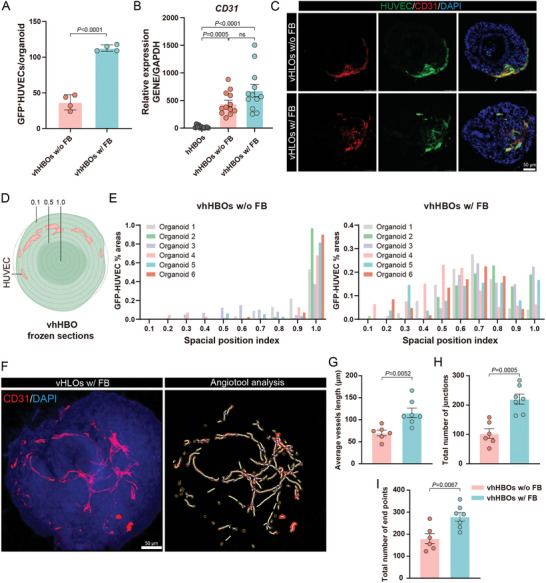
Investigation of mesenchymal grafting for vhHBOs vascularization. A) The percentage of GFP^+^HUVEC in the presence or absence of mesenchymal cells. Graphs show individual data points derived from *n* = 4 independent experiments and means ± SEM. B) qRT‐PCR analysis of the expression of endotheliocyte‐specific markers CD31 in various groups. The results are shown as the mean ± SEM of 3 independent experiments (each experiment contains 4 technical replicates). P values were calculated by unpaired two‐tailed Student's *t*‐test or one‐way ANOVA. C) Immunostaining for CD31‐expressing in the vhHBOs. D) Schematic of the methodology for calculating the relative position of GFP^+^HUVECs within the vhHBO. The whole tissue was divided into ten regions, and the average fluorescence intensity of GFP^+^HUVECs in each region was calculated. E) Representative models were compared and analyzed for percentage GFP^+^HUVECs. F) Maximum intensity projection and cross sections of a vascularized human liver organoid stained against CD31 (red) and nuclei (blue). Analysis of the corresponding projections by Angiotool, where thick white lines represent vessel skeleton, blue dots represent vessel branching points, and yellow lines highlight the outer edges of the vascular outline. Scale bars, 50 µm. G–I) AngioTool analysis of average vessel lengths, number of vessel endpoints, and number of vessel junctions based on CD31 immunofluorescence staining of vhHBOs w/o FB and vhHBOs w/ FB. Graph represents mean ± SEM of *n* ≧ 6 biological replicates from *n* = 3 independent experiments. P values were calculated by unpaired two‐tailed Student's *t*‐test.

We conducted further research to explore how mesenchymal cells enhance the stability and complexity of the vascular network. For this purpose, we quantified the average fluorescence intensity (Figure S5A, Supporting Information), the amount of GFP^+^HUVEC (**Figure** **3**A), and the expression level of the endothelial marker *CD31* (Figure 3B) on day 24. We found that the vhHBOs w/ FB group had significantly higher expression of *CD31* and higher GFP fluorescence intensity and amount. In particular, the *CD31* expression increased from 444.4 ± 61.04% in “vhHBOs w/o FB” group to 680.9 ± 111.9% in “vhHBOs w/ FB” group at day 24, this is a 23‐fold and 35‐fold increase, respectively; the fluorescence intensity of GFP^+^HUVEC has a similar trend, with the average fluorescence intensity on day 24 increasing from the “vhHBOs w/o FB” group to “vhHBOs w/ FB” increased by 1.1‐fold. These results indicate that mesenchymal cells increase the survival of endothelial cells.

To further characterize vascularization and the function and phenotypes of vhHBOs, we analyzed the positional relationships between GFP^+^HUVECs and hHBOs. We found that in the presence of fibroblast, GFP^+^HUVECs entered and expanded throughout the organoid parenchyma, presenting a luminal structure. In contrast, in its absence, GFP^+^HUVECs were predominantly distributed in the periphery of the organoids (Figure 3C–E). Furthermore, we visualized a formation of the vascular network by 100 µm‐thick organoid section staining with antibodies against endothelial cell‐specific CD31 proteins followed by fluorescence confocal microscopy (Figure 3F; Video S1, Supporting Information). We quantified the vascularization of vhHBOs generated by grafting with endothelial cells with or without mesenchymal cells using AngioTool software. AngioTool analysis revealed that the grafting of mesenchymal cells significantly increased the average vessel length, number of vessel endpoints, and number of vessel junctions in vhHBOs (Figure 3F–I). These results indicate that developing liver organoids coculture with endothelial cells and mesenchymal cells significantly improved the homogeneity of the final tissues and possessed high angiogenic potential.

### 2.5 vhHBOs Recapitulate Human Liver Cell Type, Organization, and Function

**Figure 4:**
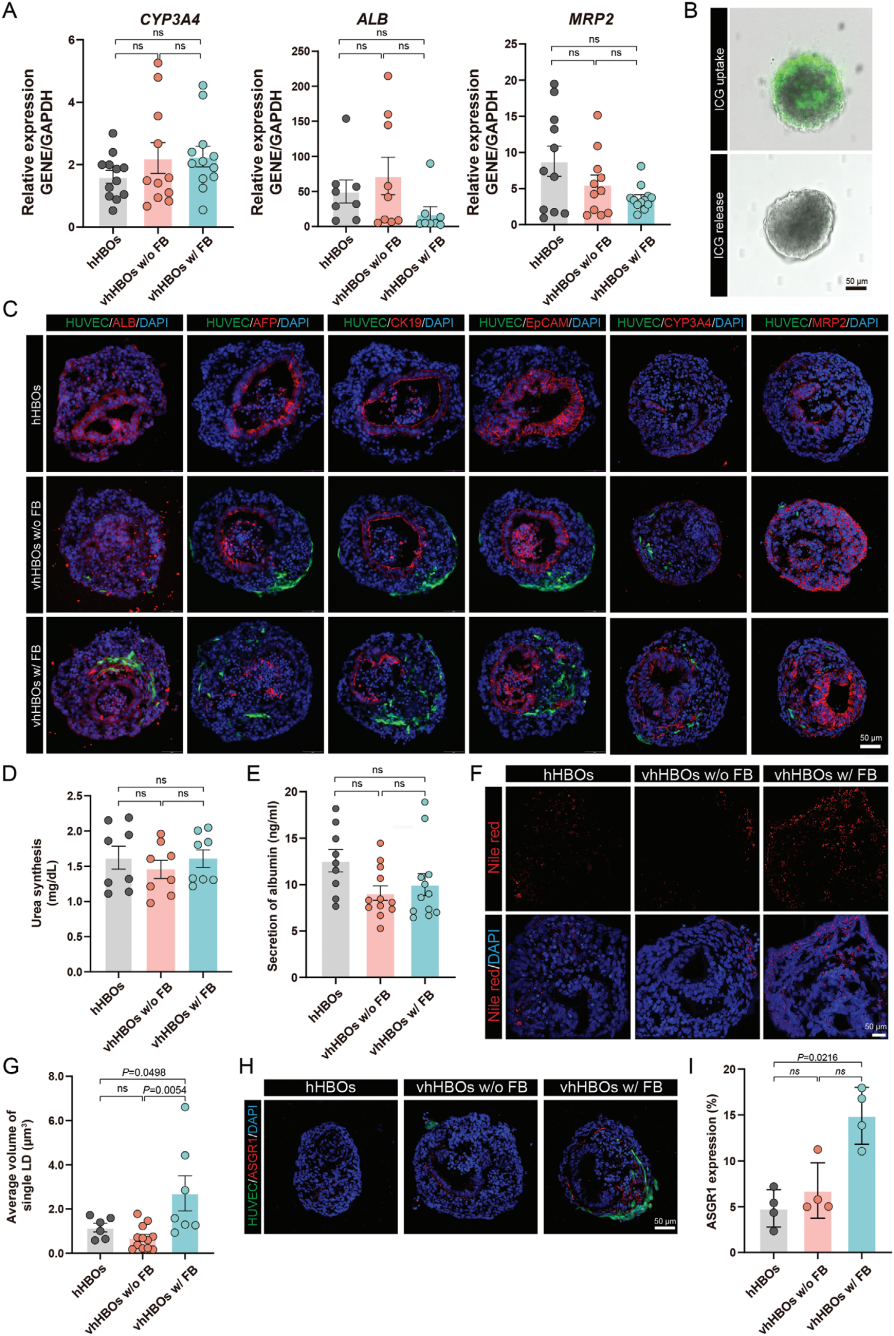
Characterization of vhHBOs. A) qRT‐PCR analysis of the expression of hepatocyte‐specific markers on day 24 under the indicated growth condition. The results are shown as the mean ± SEM of 3 independent experiments (each experiment contains 4 technical replicates). P values were calculated by unpaired two‐tailed Student's *t*‐test or one‐way ANOVA. B) ICG uptake and release of vhHBOs. Scale bars, 50 µm. C) Immunostaining for ALB, AFP, CK19, EpCAM, CYP3A4, and MRP2 in hHBOs, vhHBOs w/o FB, and vhHBOs w/ FB groups. Scale bar, 50 µm. The D) urea and E) albumin secretion in hHBOs, vhHBOs w/o FB, and vhHBOs w/ FB groups. The results are shown as the mean ± SEM of 3 independent experiments (each experiment contains 4 technical replicates). P values were calculated by unpaired two‐tailed Student's *t*‐test or one‐way ANOVA. F) Nile Red staining for lipid droplets in hHBOs, vhHBOs w/o FB, and vhHBOs w/ FB. Scale bars, 50 µm. G) Quantification of the average volume of lipid droplets. Graph represents mean ± SEM of *n* ≧ 6 biological replicates from *n* = 3 independent experiments. P values were calculated by unpaired two‐tailed Student's *t*‐test or one‐way ANOVA. H) Immunostaining for ASGR1‐expressing in the hHBOs, vhHBOs w/o FB, and vhHBOs w/ FB. Scale bars, 50 µm. I) Quantification of the proportion of ASGR1‐positive cells. Graph represents mean ± SEM of *n* = 4 biological replicates from *n* = 3 independent experiments. P values were calculated by unpaired two‐tailed Student's *t*‐test or one‐way ANOVA.

We determined that endothelial and mesenchymal cells can grow in a differentiation medium of liver organoids without adding nonparenchymal culture medium and growth factors. This discovery forms the basis for establishing vhHBOs through grafting endothelial and mesenchymal cells at various stages of liver organoid differentiation. We next determine whether vhHBOs can recapitulate human liver cell type, organization, and function. On day 18 of hHBOs differentiation, we grafted endothelial cells with or without mesenchymal cells, followed by subsequent culture for 6 days. The gene expression analysis confirmed that the expression of critical mature liver marker genes (*ALB*, *CYP3A4*, and *MRP2*) was not statistically different between all vhHBOs generated by combination with endothelial cells with or without mesenchymal cells (**Figure** **4**A). Immunostaining displayed a similar expression pattern, including the hepatocyte markers ALB, CYP3A4, MRP2, the early liver marker AFP, and the hepatic ductal cell markers CK19 and EpCAM (Figure 4C). The ALB secretion and urea synthesis showed no significant difference between group vhHBOs w/ FB and the hHBOs group (Figure 4D,E). In addition, indocyanine green (ICG) uptake and release assays demonstrate that vhHBOs were capable of taking up and releasing ICG (Figure 4B). These data also indicated the functional maturation of vhHBOs generated by combination with endothelial cells with or without mesenchymal cells. These results suggested that mesenchymal cells do not influence hHBO maturation, which permits hepatogenesis.

Moreover, we stained hHBOs with Nile red to visualize lipid droplets. We found extensive lipid accumulation within vhHBOs w/ FB (Figure 4F,G). Consistently, we found that ASGR1 was upregulated in condition “vhHBOs w/ FB”. Immunofluorescence characterization revealed that the percentage of ASGR1 expressing cells increased from 4.821% ± 1.017% in mono‐culture with only hHBOs to 6.754% ± 1.507% in condition “vhHBOs w/o FB” and to 14.90% ± 1.549% in condition “vhHBOs w/ FB” (Figure 4H,I). These results implied that grafting endothelial cells and mesenchymal cells to hHBOs improved liver lipid metabolism.

### 2.6 Transcriptional Profile of the vhHBOs

**Figure 5:**
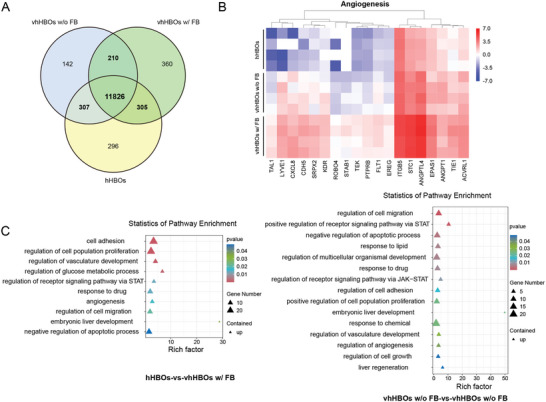
Transcriptome profile analysis of the vhHBOs. A) Venn diagram of DEG comparison showing hHBOs, vhHBOs w/o FB versus, and vhHBOs w/ FB. B) Volcanic map showing differential expression of angiogenesis‐related genes of hHBOs, vhHBOs w/o FB, and vhHBOs w/ FB groups by RNA‐seq (n = 4). C) Gene ontology (GO) terms involved in “biological process” and “molecular function” for differentially upregulated genes in the vhHBOs w/o FB and vhHBOs w/ FB groups relative to the hHBOs.

Next, we performed RNA sequencing (RNA‐seq) to comprehensively assess the changes in transcriptomic profiles of vhHBOs. Venn diagram analysis of overlapping genes indicates nonparenchymal cell grafting does not significantly change hepatic gene expression profile in terms of a small number of DEGs between vhHBOs w/o FB and hHBOs groups, and between vhHBOs w FB and vhHBOs w/o FB groups (**Figure** **5**A). Furthermore, we analyzed of upregulated differentially expressed genes. Heatmap and gene ontology (GO) enrichment analysis demonstrates enhanced vasculature development and angiogenesis in the vhHBOs w FB compared to hHBOs (Figure 5B,C). Additionally, GO enrichment analysis displays the upregulation of cell migration in the vhHBOs w FB compared to vhHBOs w/o FB. This result further supports that mesenchymal grafting promotes endothelial cells invading and sprouting into the liver organoids.

### 2.7 vhHBOs Display Hepatic and Bile Duct Zone In Vivo

**Figure 6:**
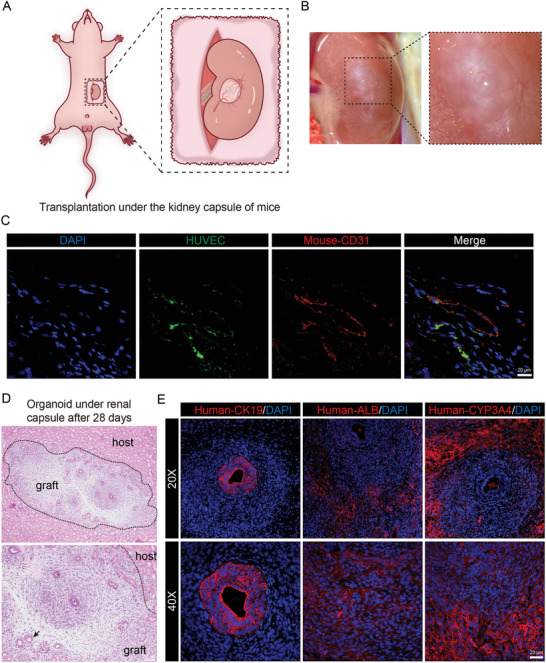
Assessment of vhHBO transplantability. A) Schematic diagram of our transplantation strategy. B) Macroscopic observation of transplanted vhHBOs showing neovascularization on the graft surface. C) Integration of HUVECs (green) and mouse CD31 (red) shown via immunofluorescence staining of paraffin section Scale bar, 20 µm. D) HE staining of transplants on day 14, with a black dotted line marking the boundary between the kidney parenchyma and the organoid. Scale bar, 50 µm. E) Immunostaining of mouse kidney/organoid transplant paraffin sections demonstrates the retention of human hepatic populations (CK19, ALB, and CYP3A4) and structures seen in vitro. Scale bar, 20 µm.

We next sought to demonstrate the potential of our vhHBOs w/ FB for clinical transplantation (**Figure** **6**A,B). These organoids were transplanted onto the left kidney capsule of mice, and after four weeks, the kidneys harboring vhHBOs w/ FB were harvested for analysis. Histological examination revealed that the engrafted organoids had developed into structures resembling hepatic cords, bile ducts, and vascular lumens typical of adult liver tissue (Figure 6C–E). This finding indicates that the vhHBOs w/ FB underwent further maturation post‐transplantation. To visualize cell types, we then performed immunofluorescence staining on sections from the grafts to assess the presence and maintenance of hepatic cell types after transplantation. Consistent with expectations, we also observed the bile duct formed that was composed of CK19^+^ALB^−^CYP3A4^−^ cholangiocytes within the ALB^+^CYP3A4^+^CK19^−^ hepatocytes in the grafts (Figure 6E). By contrast, transplantation of hHBO without endothelial cells only resulted in the appearance of ALB^−^CK19^+^ cholangiocytes and did not promote the development of ALB^+^CK19^−^ hepatocytes (Figure S6, Supporting Information). On the other hand, the formation of functional vessels was essential for determining the success of transplantation. We observed the presence of a lumen consisting of GFP^+^HUVECs and mouse CD31^+^ endothelial cells, suggesting that the preexisting GFP^+^HUVECs in the engrafted organoids had the ability to connect with host vasculature (Figure 6C). This further proves that vascularization exists within engrafted organoids and that neovascularization is ongoing.

## 3 Discussion

Currently, several strategies have been developed to pre‐vascularize organoids before organoid implant in vivo. For example, assembloid strategy explores the fusion of vascular organoids with ectoderm organoids to form pre‐vascularized ectoderm organoids such as brain organoids,^[^ ^23^, ^24^, ^25^, ^26^ ^]^ as vascular organoids usually have a comparable millimeter‐scale size with ectoderm organoids. Additionally, co‐differentiation strategy has been demonstrated to pre‐vascularize mesoderm organoids such as kidney organoids as vascular tissues belong to mesoderm and vasculogenesis usually co‐occurs with mesoderm organoid differentiation.^[^ ^27^, ^28^ ^]^ Considering the endoderm origin and relatively small size of liver organoids compared to vascular organoids, nonparenchymal cell grafting provides a new competitive route for pre‐vascularizing liver organoids.

We present the pre‐vascularization of hHBOs using intrinsic properties of nonparenchymal cells without supplementing nonparenchymal cell culture medium and essential growth factors. By grafting a number of nonparenchymal cells at the specific differentiation stage of hHBOs, endothelial cells invaded and self‐organized in the hHBOs and formed pre‐vascularized structures, enabling the following organoid transplant and fusion in vivo. Compared to the previous literature, our strategy ensures better liver parenchymal specification and ensures that liver parenchymal cells are not affected by nonparenchymal cell supplements. We explored the varied differentiation stages of hHBOs and identified the hepatic maturation stage for endothelial grafting to achieve optimal vascularization efficiency. We think that nonparenchymal cell grafting with other endothelial types (e.g., iPSC‐derived ECs or EC progenitors) may request different differentiation stage to achieve optimal grafting efficiency.

Specifically, we first investigated the optimal time for endothelial grafting during liver organoid differentiation. We identified that nearly no HUVECs integrated with the liver organoids if the endothelial grafting was conducted at the stage of hepatic specification (D10). However, the grafting efficiency for HUVECs was significantly increased from D10 to D18 with the maturation of liver organoids. We believe the optimal grafting time depends on endothelial cells' stemness. Less maturated endothelial cells, such as endothelial progenitors, may obtain an optimal grafting efficiency at the earlier stage of liver organoid than HUVECs. Additionally, the liver organoids grafted with endothelial cells can be classified into HUVEC contacted organoids or HUVEC sprouting organoids based on the phenotypes of integrated endothelial cells. The fraction of HUVEC sprouting organoids increased over the liver differentiation, indicating maturation of liver epithelium promoting endothelial sprouting. Previous research also demonstrated that developing hepatoblast secrete vascular endothelial growth factor (VEGF) and angiopoietins 1 and 2.^[^ ^29^, ^30^, ^31^ ^]^ The growth factors play critical roles in inducing enhanced vascular sprouting and promoting vascular stabilization.^[^ ^32^ ^]^ In parallel, hepatocyte growth factor (HGF) supplemented in the liver differentiation medium at liver maturation stage may also promote HUVEC sprouting phenotype for endothelial grafting on D14 and D18.^[^ ^33^ ^]^

The role of mesenchymal and endothelial interactions in vascularization is not fully understood, despite some studies demonstrating that the mesenchymal cell‐derived matrix proteins and cytokines promoted endothelial cell sprouting and lumen formation.^[^ ^34^ ^]^ We investigated the effects of mesenchymal grafting on liver organoid vascularization and found that MRC‐5, an immortalized human fetal lung fibroblast cell line, enhanced endothelial invasion into liver organoids. Previous studies have discovered that MRC‐5 cells exhibited several critical properties of human umbilical cord‐derived mesenchymal stem cells.^[^ ^35^ ^]^ Interestingly, we find that MRC‐5 grafting increases the ASGR1 expression in liver epithelium and promotes hepatic lipid accumulation. Similarly, Camp et al. reported liver bud formation by coculturing iPSC‐derived human hepatic endoderm (HE) with supportive mesenchymal and endothelial cells. Nonparenchymal cells induced overexpression of lipid metabolism‐related genes in the liver buds.^[^ ^36^ ^]^ In parallel, ASGR1 has been reported to be exclusively expressed in the liver and modulates lipid homeostasis; ASGR1 deficiency leads to the reduction of lipid droplet accumulation in the liver.^[^ ^37^ ^]^ These results revealed a potential mechanism for nonparenchymal cells to promote lipid metabolism in the iPSC‐derived hepatocytes by inducing upregulation of ASGR1 expression. Of note, except for aspects of lipid metabolism, vhHBOs w/ FB exhibit resembling key features of hHBOs, including gene expression pattern, secretion of ALB, and urea synthesis, suggesting that the endothelial and mesenchymal grafting did not affect liver fate specification.

The nonparenchymal grafting strategy enables a more homogeneous liver organoid population critical for regenerative medicine. The nonparenchymal grafting strategy enables the formation of a uniform liver organoid population critical for regenerative therapy. We discovered that MRC‐5 reduced the phenotypic heterogeneity of vhHBOs in their size and circularity. Additionally, we excluded chemically undefined components, such as Matrigel (widely used in liver organoid differentiation) and serum (widely used in nonparenchymal cell culture) in the liver differentiation process to reduce variation. In addition, using a mHCPCA device also ensures the reproducibility of organoid formation compared to the conventional Matrigel dome method.^[^ ^38^, ^39^, ^40^, ^41^ ^]^ Moreover, mHCPCA enables to generate 8000 vhHBOs (≈10^9^ cells) in a 48‐well plate each time, which can fully fill the cell quantity demand for cell therapy.^[^ ^42^, ^43^ ^]^ Thus, this NCG strategy together with mHCPCA‐based organoid production device is ready for cell therapy, such as end‐stage liver diseases, acute liver failure, and bile duct repair after liver transplantation.

Finally, vhHBOs demonstrate outstanding potential for simultaneously repairing hepatic tissue and bile duct damage. Ectopic vhHBO implants into mice displayed integration of vhHBO with native tissue and interconnection of vascular networks. Notably, transplanted vhHBOs self‐organized into native liver tissue like hepatic zone (CK19^−^ALB^+^CYP3A4^+^) and bile ducts (CK19^+^ALB^−^CYP3A4^−^). We believe that the vascular networks in the vhHBO transplanted regions are functional as the tissue architecture and cell nuclei morphology indicate cells in these regions were viable 4 weeks post transplantation. Notably, we found for the first time in the organoid field that the transplanted vhHBOs self‐organized into intrahepatic bile ducts surrounded by hepatic tissues, indicating their potential to cure liver damage in both hepatic tissues and intrahepatic bile ducts.

In conclusion, we present a promising strategy for generating vhHBOs via endothelial and mesenchymal grafting in a Matrigel‐free and nonparenchymal growth factor‐free culture environment. The vhHBOs, with their hierarchically organized liver parenchyma cells and sprouting endothelial cells, can be produced in large quantities. The uniform size of vhHBOs and their bipotent capacity to repair both hepatic tissues and bile ducts make them a hopeful solution for liver and bile duct injuries in liver transplants. We believe this NCG strategy has the potential to be universal for vascularizing other organoid types, opening up new possibilities in tissue engineering and regenerative medicine.

## 4 Experimental Section

### Fabrication of mHCPCA Chips

The mHCPCA chips were fabricated via micro‐molding as previously described.^[^ ^21^ ^]^ Specifically, using the CNC micro‐milling method, we engraved microwells in polymethyl methacrylate (PMMA) plates at equal distances. PDMS (1, 10 mixture of cross‐linking agent with elastomer) was poured onto the PMMA female mold and placed in a vacuum chamber for 1 h to remove air bubbles, then cured at 80 °C overnight. After cross‐linking, the PDMS stamps were carefully released from the PMMA female mold. The 2% (w/v) agarose solution was deposited onto the PDMS stamp and incubated at 4 °C for 20 min for gel formation. After gelation, the micropatterned agarose scaffold was released and punched with the appropriate diameters. Finally, the desired mHCPCAs were placed at the bottom of the wells in a 48‐well plate, thoroughly sterilized under UV light, and stored at 4 °C for subsequent use. Each array contained 170 microcavities of 500 µm in diameter and 1 mm in depth, which were tightly arranged in a triangular shape at 200 µm intervals.

### hiPSCs Culture and Foregut Differentiation

hiPSCs were maintained on vitronectin (VTN)‐coated dishes in Nuwacell ncTarget hPSC Medium (Nuwacell Biotechnologies, Hefei, China). hiPSCs were differentiated into foregut using a previously described method with minor modifications.^[^ ^21^, ^44^, ^45^ ^]^ In brief, hiPSCs were detached using Accutase (Gibco, Rockville, USA) and seeded on VTN‐coated tissue culture plates at a seeding density of 100 000 cells per cm^2^. When the cells reached 85%–90% confluence, the culture medium was replaced with RPMI 1640 medium containing 100 ng mL^−1^ Activin A (CELL guidance systems, Cambridge, UK) and 50 ng mL^−1^ BMP4 (R&D Systems, Minneapolis, USA) at day 1, 100 ng mL^−1^ Activin A and 0.2% knockout serum replacement (Gibco, Rockville, USA) at day 2, and 100 ng mL^−1^ Activin A and 2% knockout serum replacement at day 3. On days 4–6, the cells were cultured in advanced DMEM/F12 with 1% B27 (Thermo Fisher, Waltham, USA) and 1% N2 (Thermo Fisher, Waltham, USA) containing 500 ng mL^−1^ FGF2 (Source Bioscience, Nottingham, USA) and 3 µm CHIR99021 (Stemgent, Beltsville, USA). All cell cultures were maintained at 37 °C with 5% CO_2_ and 95% air, and the medium was replaced daily.

### hHBO Induction

At day 6, foregut cells were gently detached into single cells using Accutase. They were subsequently washed with advanced DMEM/F12. After centrifugation at 1000 rpm for 4 min at 4 °C, the cells were resuspended at the appropriate density in liver organoid formation medium comprising advanced DMEM/F12 containing 1% B27 (Thermo Fisher, Waltham, USA), 1% N2 (Thermo Fisher, Waltham, USA), 80 ng mL^−1^ FGF2 (Source Bioscience, Nottingham, USA), 3 µm CHIR99021 (Stemgent, Beltsville, USA) and 10 µm Y27632 to deposit typically 200 cells per microwell. After organoid formation, the culture medium was switched to the liver organoid specification medium comprising advanced DMEM/F12 containing 1% B27 (Thermo Fisher, Waltham, USA), 1% N2 (Thermo Fisher, Waltham, USA), and 2 µm retinoic acid (RA; Sigma, St. Louis, USA) for 4 days. After RA treatment, the medium was changed to liver maturation media comprising Hepatocyte Culture Medium (HCM; Lonza, Basel Stücki, Switzerland) with 10 ng mL^−1^ HGF (PeproTech, USA), 20 ng mL^−1^ OSM (R&D Systems, Minneapolis, USA), and 100 nM Dexamethasone (Sigma, St. Louis, USA) for 10 days. Organoid induction and culture were performed at 37 °C with 5% CO_2_, 95% air and the full medium was replaced every 2–3 days.

### Endothelial and Mesenchymal Cell Culture

Green fluorescence protein tagged human umbilical vein endothelial cells (GFP^+^ HUVECs) were cultured on 0.1% gelatin (Sigma, St. Louis, USA) coated dishes in Endothelial Cell Media (ScienCell, Carlsbad, USA). MRC‐5 was cultured in MEM medium (Bosterbio, Wuhan, China) supplemented with 10% (vol/vol) fetal bovine serum (Bio‐One, Shanghai, China) and 1% (vol/vol) penicillin/streptomycin (Gbico, Rockville, USA). All cell lines were used between passages 5–10 and maintained in a humidified incubator at 37 °C and 5% CO_2_.

### Grafting Endothelial Cells to hHBOs

At multiple time points during differentiation (day 10, day 14, day 18), GFP^+^HUVECs were dissociated into single cells using 0.5% TrypLE Express (Gibco, Rockville, USA) for 1 min at 37 °C. After centrifugation at 1000 rpm for 4 min at 4 °C, GFP^+^HUVECs were resuspended in liver organoid specification medium or liver maturation media. Freshly GFP^+^HUVECs suspension was then seeded at the appropriate density (typically 670 GFP^+^HUVECs per microwell) on top of a micropatterned agarose scaffold filled with hHBOs. GFP^+^HUVECs were allowed to sediment, and organoids containing GFP^+^HUVECs were detected 24 h later.

### Grafting Endothelial and Mesenchymal Cells to hHBOs

At day 18, during differentiation, GFP^+^HUVECs and MRC‐5 cells were dissociated into single cells using 0.5% TrypLE Express (Gibco, Rockville, USA) for 1 and 3 min at 37 °C, respectively. After centrifugation at 1000 rpm for 4 min at 4 °C, these two cells were then mixed in liver maturation medium at the following two endothelial‐to‐fibroblast cell ratios of 5, 0 and 5, 2. In the 5, 0 ratio, 170 000 GFP^+^ HUVECs without MRC‐5 cells (typically 1000 GFP^+^ HUVECs per microwell) were used as coculture. In the 5, 2 ratio, 170 000 GFP^+^ HUVECs and 68 000 MRC‐5 cells (typically 1000 GFP^+^ HUVECs and 400 MRC‐5 cells per microwell) were used as grafting. After mixing, the fresh cell suspension was then seeded on top of a micropatterned agarose scaffold filled with hHBOs as described above. The angiogenic sprouting formation efficiency was assessed on day 7 after seeding.

### Gene Expression Analysis

Total RNA was extracted from organoids using a Trizol (Sheng Gong, Shanghai, China) reagent. Reverse transcription was performed with ABScript II RT Master Mix for qPCR (ABclonal, Wuhan, China) following the manufacturer's instructions. Real‐time PCR was performed using SYBR Green Real‐Time PCR Master Mixes (ABclonal, Wuhan, China) under 40 cycles with the following conditions, denaturation at 95 °C for 1 min, annealing at 58 °C for 30 s, and extension at 72 °C for 30 s. Gene‐specific primer sequences were designed using the NCBI Primer Design Tool and listed in Table S1 (Supporting Information). All genes were normalized to *GAPDH* and assessed relative to the expression levels of the un‐transduced hiPSCs. Data analysis followed the 2^−ΔΔCt^ method, and the results are representative of three independent experiments.

### RNA‐seq Analysis

P‐values were adjusted for multiple testing using the Benjamini‐Hochberg method to control the false discovery rate. Genes with an adjusted P‐value < 0.05 and a fold change ≥2 were classified as differentially expressed. Gene ontology (GO) enrichment analysis was performed using the DAVID database, focusing on DEGs with a fold change ≥2 to ensure a stringent dataset. RNA sequencing and data analysis were supported by Seqhealth Technology Company Limited.

We collected these 3 groups (hHBOs, vhHBOs w/o FB, and vhHBOs w/ FB groups) of organoids and extracted RNA on the 24th day of cultivation (*n* = 4). For each biological replicate, about 170 liver organoids were used for RNA‐seq. RNA quality control was conducted to ensure purity, concentration, and integrity. RNA sequencing was performed on the Illumina NextSeq 6000 platform, generating an average of 20 million reads per sample. Differential expression analysis between groups was conducted using DESeq2, which employs a negative binomial distribution model to identify differentially expressed genes (DEGs) in digital gene expression data. Genes with an adjusted P‐value < 0.05 and a fold change ≥2 were classified as differentially expressed. Gene ontology (GO) enrichment analysis was performed using the DAVID database, focusing on DEGs with a fold change ≥2 to ensure a stringent dataset. The transcriptomics data have been submitted to the Sequence Read Archive (database identifier, PRJNA1196083).

### Immunofluorescence (IF)

Organoids and tissue were fixed in 4% (w/v) paraformaldehyde at 4 °C overnight. Sections were prepared and subjected to immunofluorescence staining and H&E. The thickness of the sections was 8 µm, except for the section used in Figure 4B, which was 100 µm. For paraffin sections, the paraffin was first deparaffinized in xylene, rehydrated with a graded series of ethanol, and boiled in 10 mm sodium citrate buffer solution. For frozen sections, standard immunofluorescence protocol was performed directly. The slides were washed in PBS, followed by 15 min permeabilization at 0.5% Triton X‐100 in PBS. Subsequently, the slides were washed thrice in PBS for 5 min and blocked for 60 min in 1% (v/v) BSA and 0.5% Triton X‐100 in PBS. The slides were incubated with primary antibodies at 4 °C overnight. After washing with PBS, slides were incubated with the secondary antibodies at room temperature for one hour. All the antibodies used in this study are listed in Table S2 (Supporting Information). Images were captured on a fluorescence microscope (Olympus IX‐83; Leica TCS SP8 STED).

### Quantification of Vessel Intensity and Organoid Size

Size and vessel intensity per organoid were analyzed using the open‐access Fiji (ImageJ) software. More particularly, the projected contours of the organoids were redrawn using an automatic identification method, and the area and circularity surrounded by their closed regions were measured using the “enclose” function. Protocols for assessing the efficiency of angiogenic sprouting were modified from previous reports.^[^ ^46^ ^]^ In brief, maximum intensity projections were acquired using the Olympus IX‐83 imaging system at 10× magnification. FIJI v.1.54f was used to quantify the relative GFP^+^HUVEC vessel intensity. Organoids and background regions were selected as regions of interest (ROI) with the blow/lasso tool, and organoid vessel intensity was acquired by subtracting the background region's average fluorescence intensity from the organoids region's average fluorescence intensity. These results represent the results of three independent experiments. All of the plots were generated using Prism 9 software. Vessel intensity was calculated using the formula.

(1) $$\begin{array}{ccc} & & \mathrm{Fluorescence}\;\mathrm{intensit}y_{\mathrm{ROI}}=\mathrm{IntD}e_{\mathrm{ROI}}\\ & & -\left(\mathrm{Are}a_{\mathrm{ROI}}\times \mathrm{Mean}\;\mathrm{fluorescenc}e_{\mathrm{background}\mathrm{readings}}\right) \end{array}$$

### Quantifying GFP^+^HUVEC Counts

Organoids were collected from the microfabricated hexagonal closely packed cavity array chips and placed in a 15 mL centrifuge tubes with each tube containing about 170 organoids. To obtained single‐cell suspensions, organoids were washed with PBS and then digested using Accutase solution (STEMCELL Technologies) at 37 °C for 30 min. Then, the suspension was collected in the PBS solution, centrifuged at 300 g for 5 min, and resuspended in 200 µL of liver maturation medium containing Propidium Iodide (Pepto‐Bismol) for counting.

### Quantifying GFP^+^HUVEC Area Fraction

To quantify the area coverage rate of GFP^+^HUVECs in the organoid tissue section, a border was first drawn around the organoid based on the DAPI channel using ImageJ software. Then GFP signals beyond the border mask removed. The area of GFP^+^ region was calculated using a cutoff threshold.

### AngioTool Analysis

Confocal z‐stacks of vhHBOs were taken from100 µm‐thick organoid section samples. Process these Z‐stacks with the Imairs 9.0.1 software to obtain the maximum intensity projections. Quantification of vessel parameters of the vhHBOs from single‐channel grayscale images of CD31 staining using AngioTool following automated protocols.^[^ ^47^ ^]^

### Albumin (ALB) and Urea Secretion Assay

To evaluate the ALB and urea secretion ability of the organoids, the supernatant culture media was collected 24 h after the last media refreshment and stored at −80 °C before beginning the assay. The concentrations of ALB in the supernatant were assessed using an enzyme‐linked immunosorbent assay (ELISA) kit specific for each protein or metabolite according to the manufacturer's instructions; specifically, the kits included the Human Albumin ELISA kit (Bethyl, E80‐129), the QuantiChrom urea assay kit (Bioassay Systems, DIUR‐100). All the samples were carried out in triplicate.

### ICG Uptake and Release Assay

Protocol for assaying the uptake and release of ICG by vhHBOs was based on previous reports.^[^ ^48^, ^49^ ^]^ Dry ICG powder (Sigma, St. Louis, USA) was dissolved in liver maturation media to obtain a 1 mg mL^−1^ ICG solution. For ICG uptake assay, the organoids were exposed to a 1 mg mL^−1^ ICG solution at 37 °C for 4 h and then washed three times in DPBS (Gibco, Rockville, USA). For the ICG release assay, the organoids were replaced with fresh liver maturation media and incubated for a further 12 h to determine the ICG release. ICG uptake and release images were captured immediately using a multiphoton microscope (Leica TCS SP8).

### Nile Red Staining

For lipid staining, organoid sections were air‐dried for 30 min, washed with PBS for three times and incubated with 0.5 µg mL^−1^ Nile Red (Sigma) and 1 µg mL^−1^ DAPI (Thermo Fisher) for 20 min at RT. Each section is mounted on a glass microscope slide and covered with a cover slip. Images were taken immediately via a 63× objective of a fluorescence microscope (Leica TCS SP8 STED) after mounting.

### Transplantation Under the Rodent Kidney Capsule

Bright‐field imaging‐based phenotype screening of vhHBO was conducted and successful differentiated vhHBO batch was identified for transplantation. In brief, successful differentiated vhHBOs displayed the phenotypic features including, 1) few dead cells and cell debris surrounding the vhHBO in the microwell; 2) well‐defined boundaries of organoid; 3) organoid size was around 300 µm. Organoids without the above features were excluded from the transplantation experiments.

All experimental procedures were approved by the institutional review board of the Institute of Organ Transplantation at Tongji Hospital (TJH‐202401025). The transplantation of the organoids under the kidney capsule or the sham laparotomy was performed as described method with some modifications.^[^ ^50^ ^]^ In vitro generated 1 × 10^6^ cell‐equivalent LBs were collected and transplanted into a kidney capsule of a non‐obese diabetic/severe combined immunodeficient (NOD/SCID) mouse. The sham group received 50 µL of sterile saline. Animals were sacrificed 4 weeks post‐transplantation, and the kidney were fixed in 10% formalin for analysis.

### Statistical Analysis

Statistical analysis was done in GraphPad Prism 9 (GraphPad Software, USA), and statistical significance was attributed to values of *P* < 0.05 as determined by Student's *t*‐test or one‐way ANOVA analysis, as described in the figure legends. Data are expressed as mean ± SEM.

## Conflict of Interest

The authors declare no conflict of interest.

## Author Contributions

P.C. was responsible for conceptualization. P.C., H.F., and J.S. performed data curation. H.F. and J.S. handled formal analysis. Funding acquisition was made by P.C., Z.C., and B.Y. Investigation was performed by H.F., J.S., J.L., and P.C. Methodology was prepared by H.F., J.S., J.L., B.Y., and P.C. Project administration was handled by P.C. Resources were arranged by P.C. and Z.C. Software was the responsibility of H.F. and J.S. Supervision was conducted by P.C. and Z.C. Validation was handled by H.F., J.S., J.L., D.Z., S.J., and P.C. Visualization was performed by H.F., J.S., Y.Z., Y.W., and P.L. Also, H.F., J.S., and P.C. wrote the original draft. Writing, review and editing were performed by P.C., C.L., H.F., J.S., and Y.F.

## Supporting information



Supporting Information

Supplemental Video 1

## Data Availability

The data that support the findings of this study are available in the supplementary material of this article.
