# Supplementary material for: High‐Throughput Formation of Pre‐Vascularized hiPSC‐Derived Hepatobiliary Organoids on a Chip via Nonparenchymal Cell Grafting
Source: Adv Sci (Weinh). 2025 Jan 4;12(8):2407945. doi: 10.1002/advs.202407945 (PMC11848576; doi:10.1002/advs.202407945)
Supplement: Supplementary file 1 — Supporting Information [file ADVS-12-2407945-s002.docx]

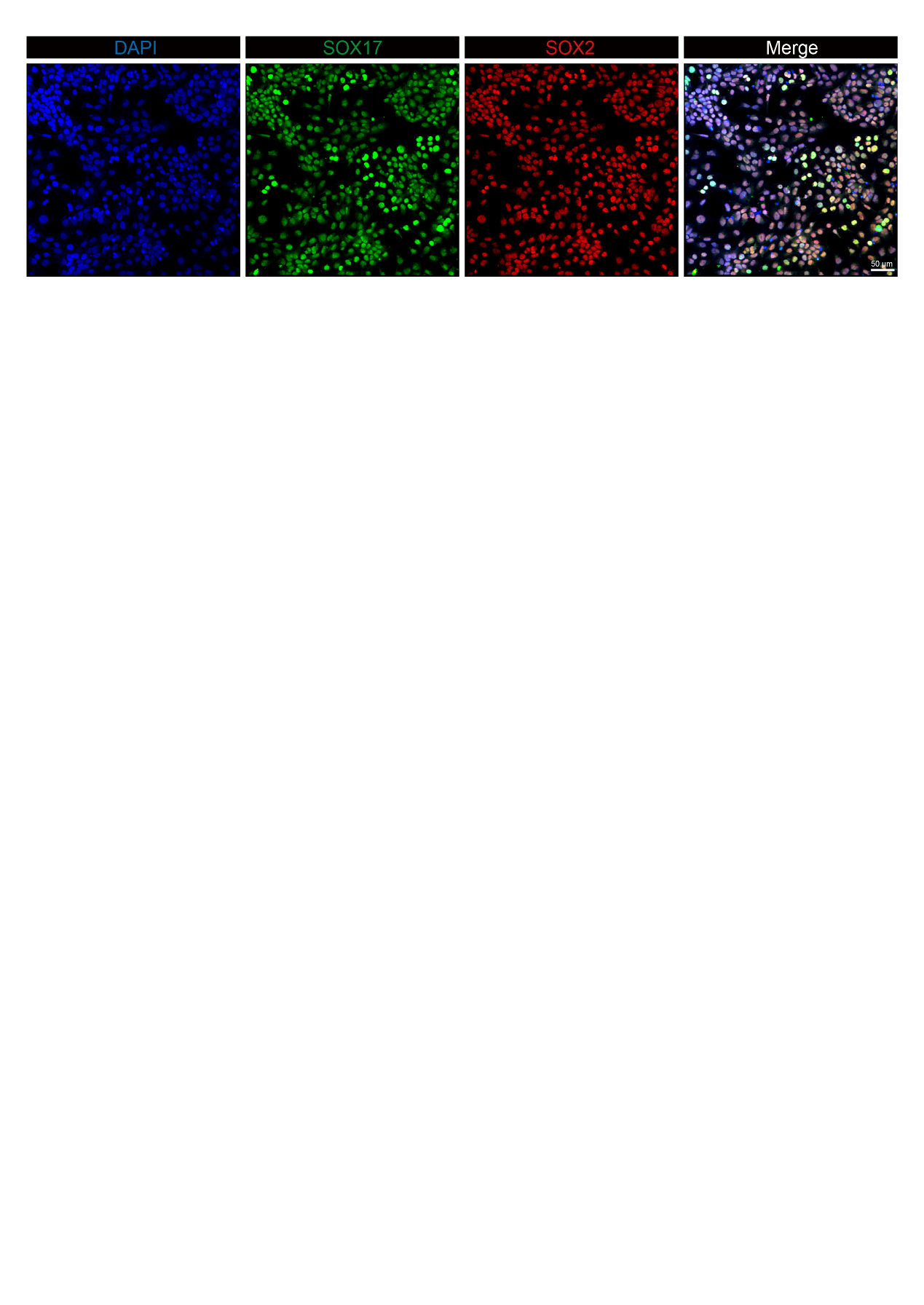


**Figure S1 Characterization of hiPSC-derived hFSCs. Immunofluorescence staining of differentiated cells with hFSC-specific markers SOX17 (green) and SOX2 (red).** Scale bars, 50 µm.


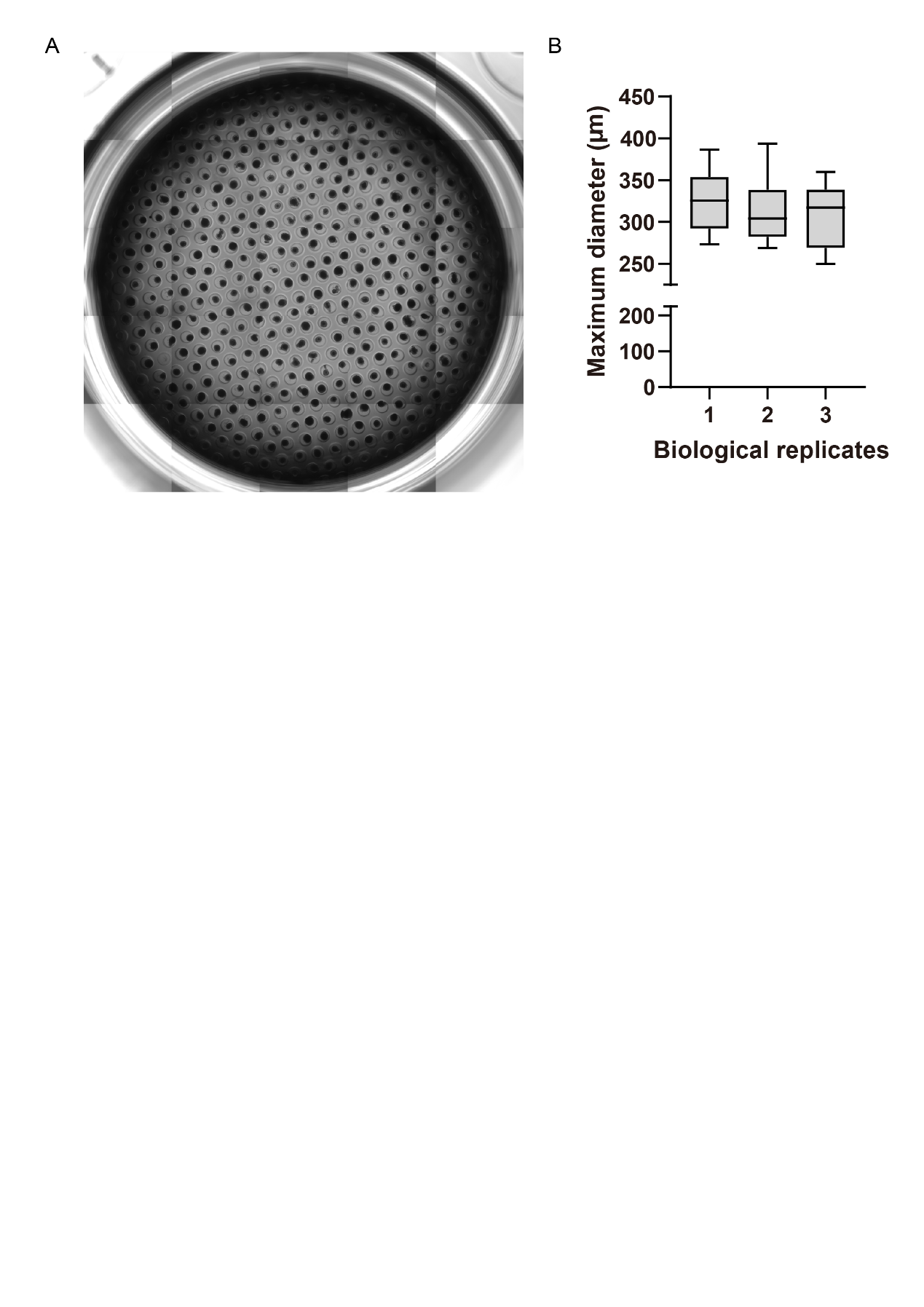


**Figure S2 Characterization of the reproducibility of hHBOs formation in mHCPCAs.**

(A) Representative bright-field images of hHBOs cultured in mHCPCAs on day 24 (B) Maximum diameter of the hHBOs in three biological replicates.


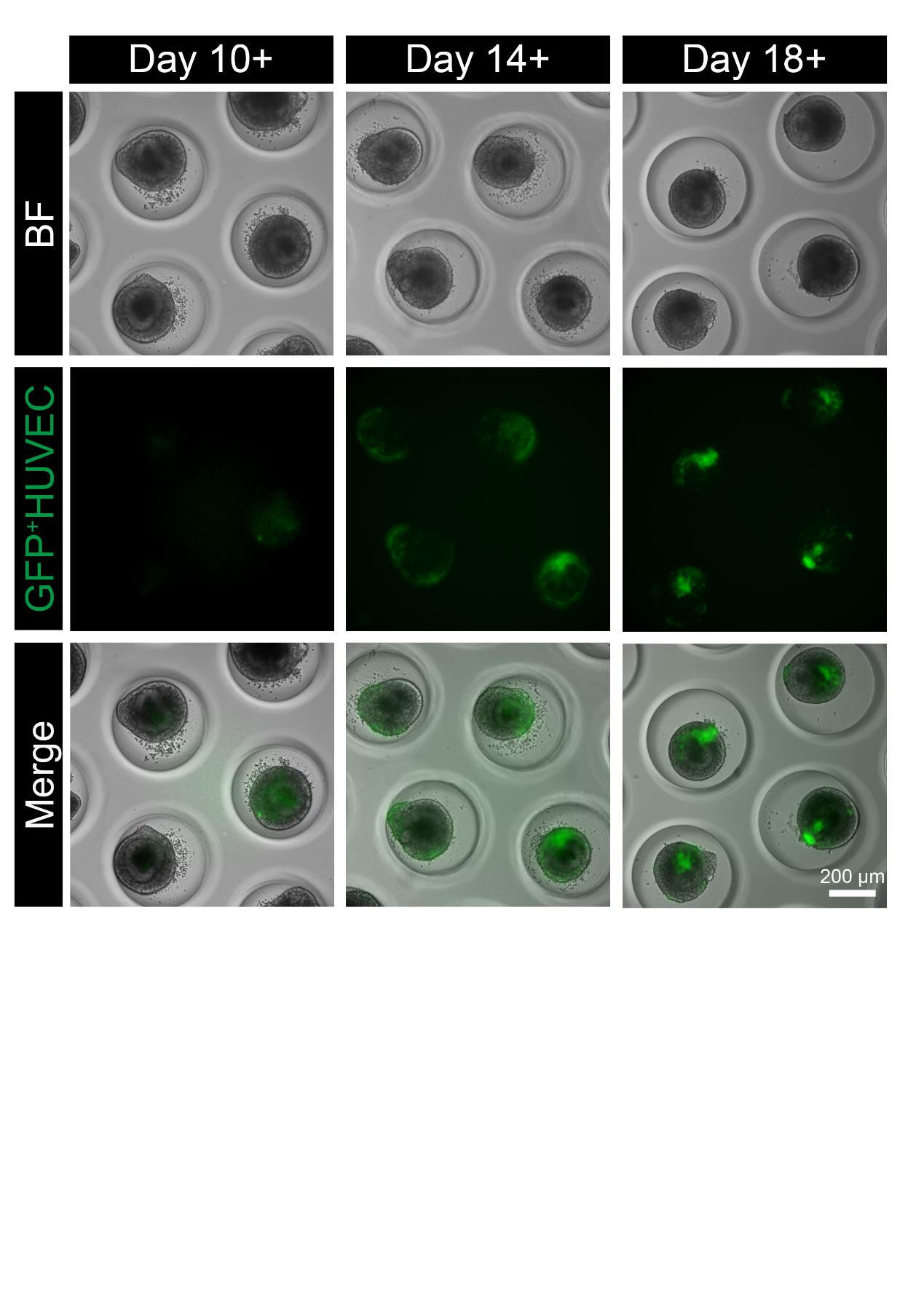


**Figure S3 Comparative morphological analysis of GFP^+^HUVEC integration with hHBOs at different stages (days 10, 14, and 18), shown through the bright field (top) and fluorescence microscopy (bottom).** Scale bar, 200 μm.


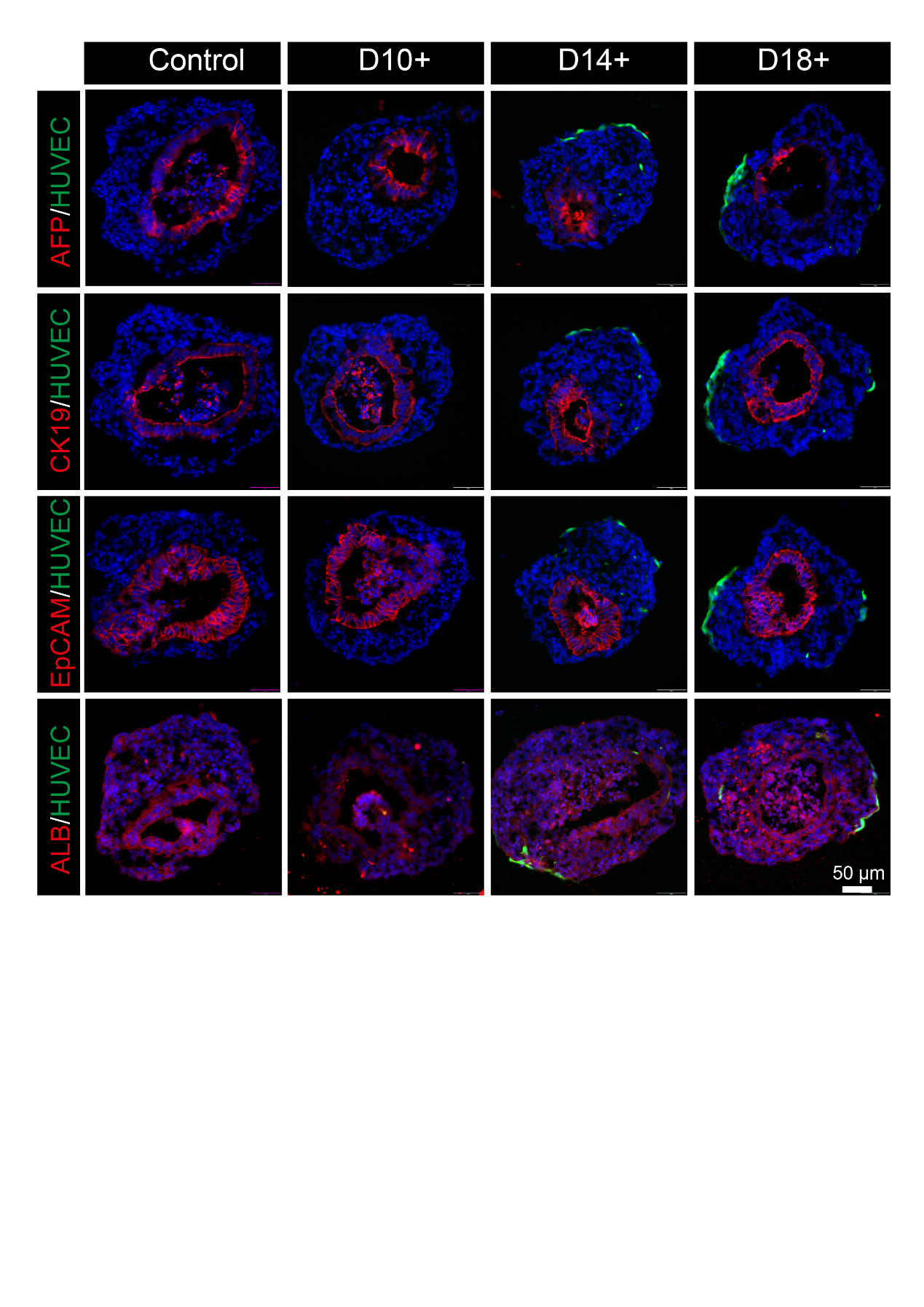


**Figure S4** **Immunostaining for hepatic markers (AFP, CK19, EpCAM, and ALB) in the hHBOs cultured alone or in combination with GFP+HUVECs (green)**. Scale bar, 50 μm.


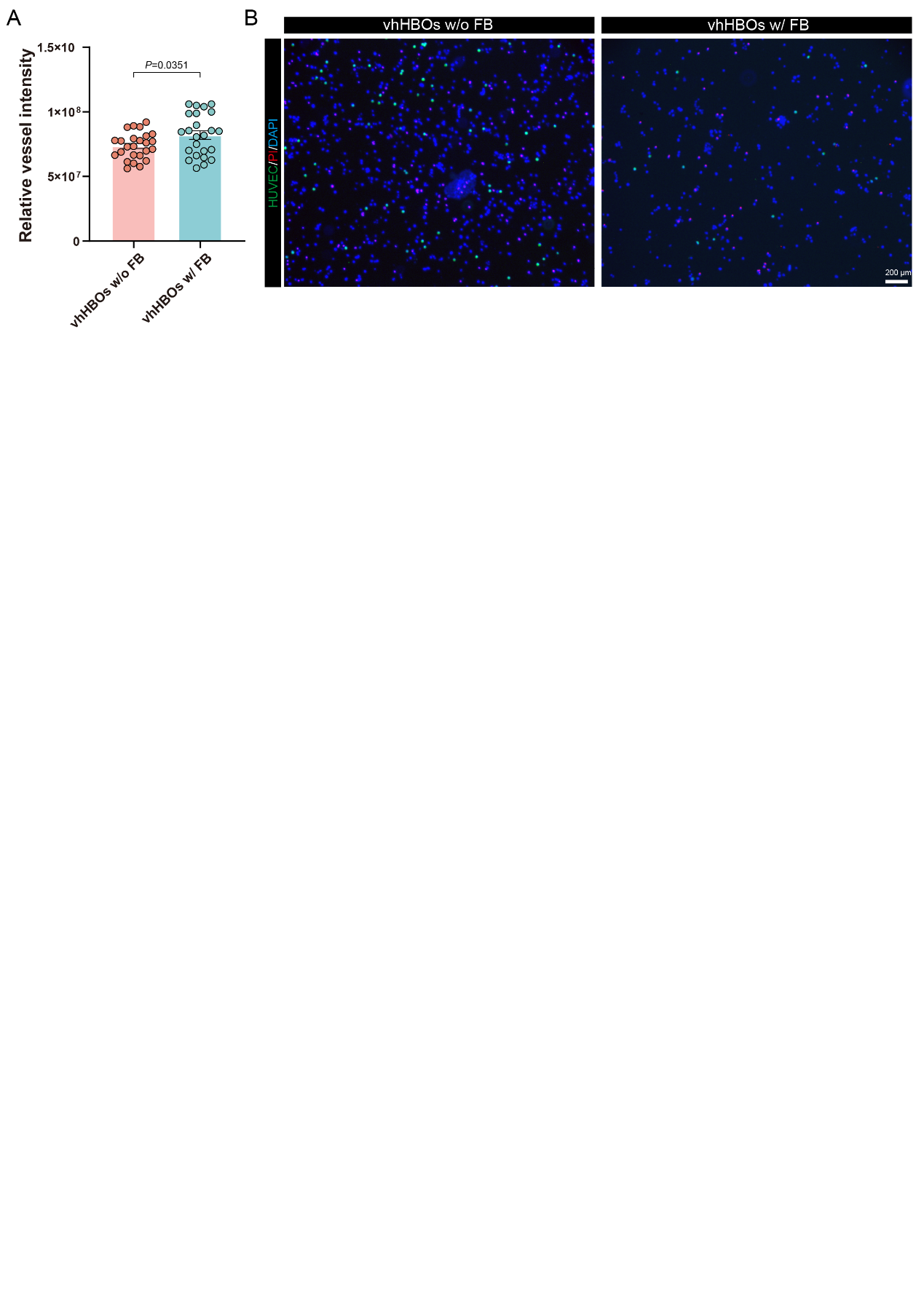


**Figure S5** **Characterize the vascularization efficiency of vhHBOs w/o FB and vhHBOs w/ FB groups.**

(A) Relative microvascular the GFP signal in the presence or absence of mesenchymal cells. Results represent mean ± SEM; n = 15–20 vhHBOs. (B) DAPI (blue) and PI (red) staining of single-cell suspensions from vhHBOs w/o FB and vhHBOs w/ FB groups, respectively.


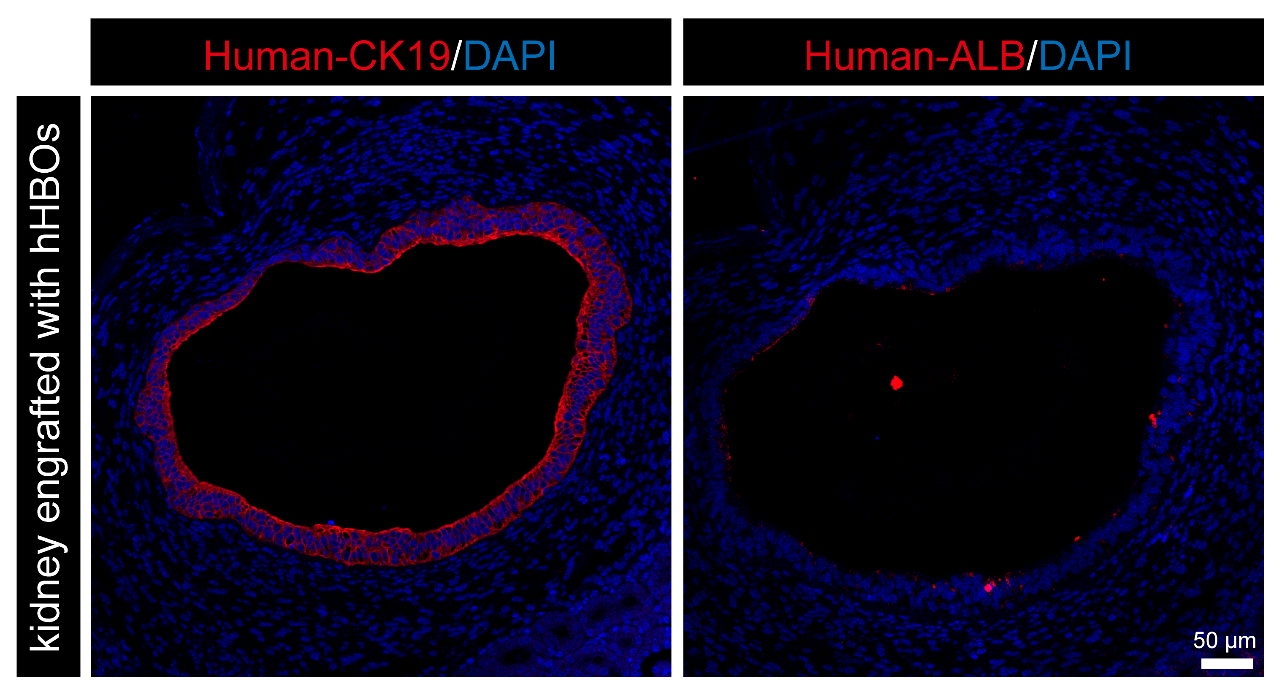


**Figure S6 Immunofluorescent analysis of human ALB and CK19 expression in the kidney engrafted with hHBOs on day 24.** Scale bar: 50 µm.

Table S1. Primer sequences used for qRT-PCR analysis

| Gene | Forward primer | Reverse primer |
| --- | --- | --- |
| *GAPDH* | GAAGGTGAAGGTCGGAGTC | GAAGATGGTGATGGGATTTC |
| *CD31* | AAGGTCAGCAGCATCGTG | AGTGCAGATATACGTCCC |
| *ALB* | TGCTGAGGCAAAGGATGTCT | ATGTCTTGGCAAGTCTCAGCA |
| *MRP2* | CCCTGCTGTTCGATATACCAATC | TCGAGAGAATCCAGAATAGGGAC |
| *CYP3A4* | CGTAAGTGGAGCCTGATTTCCCT | AATGGTGCTAACTGGGGGTGGTG |

Table S2. List of primary antibodies used in immunofluorescence microscopy

| Resource | Source | Identifier |
| --- | --- | --- |
| Rabbit anti-human AFP | Dako | Cat# A008 |
| Rabbit anti-human Cytokeratin 19 | Abcam | Cat# ab76539 |
| Mouse anti-human EpCAM | Cell Signaling Technology | Cat# 2929 |
| Goat anti-human Albumin | Bethy | Cat# A80-129A |
| Rabbit anti-human CD31 | Invitrogen | Cat# MA5-29474 |
| Mouse anti-mouse CD31 | Santa Cruz | Cat# sc-46694 |
| Mouse anti-human CYP3A4 | Santa Cruz | Cat# sc-53850 |
| Rabbit anti-human MRP2 | Abcam | Cat# ab172630 |
| Mouse anti-human ASGR1 | Santa Cruz | Cat# sc-52623 |
| Goat anti-human SOX17 | R&D Systems | Cat# AF1924-SF |
| Rabbit anti-human SOX2 | Boster | Cat# BIO-19 |
